# Brief report on a phase I/IIa study to assess the safety, tolerability, and immune response of AGMG0201 in patients with essential hypertension

**DOI:** 10.1038/s41440-021-00755-6

**Published:** 2021-10-17

**Authors:** Hironori Nakagami, Tetsuya Ishihama, Yuichi Daikyoji, Chieka Sasakura, Ei Yamada, Ryuichi Morishita

**Affiliations:** 1 Department of Health Development and Medicine, Graduate School of Medicine, Osaka University, 2-2 Yamada-oka, Suita, Osaka 565-0871 Japan; 2 AnGes, Inc., 4-13-3, Minato-ku, Tokyo 108-0014 Japan; 3 Department of Clinical Gene Therapy, Graduate School of Medicine, Osaka University, 2-2 Yamada-oka, Suita, Osaka 565-0871 Japan

**Keywords:** Angiotensin II, Hypertension, Vaccine

## Abstract

We have been developing an angiotensin II vaccine for hypertension. We conducted a placebo-controlled dose escalation study to investigate the safety, tolerability, and immunological responses of this angiotensin II vaccine (AGMG0201). AGMG0201 was administered to participants with mild to moderate hypertension between 18 and 79 years of age. Twelve patients each were enrolled in the low-dose and high-dose groups. Within each group, subjects were randomly assigned to receive either the active study drug or a placebo at a ratio of 3:1. Each participant received a single intramuscular injection, followed by a second injection 30 days later, and was monitored for 360 days after the second dose. The results showed that most treatment-related adverse events were classified as mild or moderate in severity, including pain and erythema at the injection site. Anti-angiotensin II antibodies were observed in the AGMG0201 patients, especially in the high-dose group. Overall, AGMG0201 was well tolerated.

## Introduction

Several clinical trials of vaccines for hypertension have been reported [1–5]. A double-blind, randomized, placebo-controlled phase IIa trial was conducted to investigate the effect of an angiotensin II vaccine (AngQb-Cyt006) in 72 patients with mild to moderate hypertension [5]. The high-dose group had a lower mean ambulatory daytime systolic BP than the placebo group, but work has stopped on this drug. We have been developing an angiotensin II vaccine in several animal models [6–10], and we have combined the DNA vaccine with a peptide vaccine and adjuvants for the first in human clinical trials. We conducted a double-blind, randomized, placebo-controlled phase I/IIa trial with a modified angiotensin II DNA vaccine (AGMG0201) to evaluate its safety, tolerability, and immune response.

## Method

### Study setting

This study is a randomized, double-blind study in adults between 18 and 79 years of age with mild to moderate essential hypertension.

### Key endpoints

#### Primary endpoints

Solicited adverse events (AEs) (local and systemic reactogenicity events) were collected for 90 days after vaccination, and unsolicited events were collected for 90 days after vaccination.

#### Secondary endpoint

The secondary outcome was the immunological response to AGMG0201 as determined by the anti-angiotensin II antibody titer measured at 7, 14, and 30 days after the first vaccination and at 7, 14, 30, 60, 90, 180, and 360 days after a booster vaccination.

### Key inclusion criteria

1. Participants had to have mild to moderate hypertension at either of the screening visits, defined as a mean systolic BP of 140–179 mmHg and/or a mean diastolic BP of 90–109 mmHg (inclusive) [11]. Participants who had an established regimen of oral hypertensive medication at the first screening visit (Screen 1) had to meet these criteria at a second screening visit (Screen 2) following a ≥ 14-day washout from their antihypertensive medication.
2. Participants who were not taking antihypertensive medication at the time of the first screening visit were eligible, as were participants who were on either a single antihypertensive medication regimen (including but not limited to CCBs, diuretics, ARBs, ACEIs, α blockers and β blockers) or a combined ACEi/ARB + CCB or ACEi/ARB + diuretic regimen and were willing to discontinue antihypertensive treatment from at least 14 days prior to the first vaccination to 90 days after the booster vaccination (4.5 months total).
3. All study participants were required to come to an initial screening visit (Screen 1) up to 90 days prior to enrollment (Visit V0). Depending on their oral antihypertensive use at the screening, potential subjects might have needed to undergo a run-in period of 2–4 weeks to assess their tolerance to high-dose ARBs/ACEi, followed by a second screening visit (Screen 2). All participants underwent a 2-week washout period prior to V0.

### Screening and treatment

Subjects were enrolled in two groups of 12 subjects (Supplementary Table S1): Group 1 (low dose or placebo) and Group 2 (high dose or placebo). Within each group, subjects were randomly assigned either the active study drug or a placebo at a ratio of 3:1. In both groups, two sentinel subjects initially received either the active study drug or a placebo (randomly assigned 1:1), and after a minimum of seven days and a review of the safety data by the Safety Review Committee, another two sentinel subjects received either the study drug or a placebo (randomly assigned 1:1). All available sentinel safety data, including at least 30 days of booster vaccination data from the additional sentinel subjects, were reviewed prior to administration of the study drug to the remaining 8 subjects in both groups (randomly assigned seven active drug:one placebo).

Dose administration to the first sentinel subjects in Group 2 did not commence until all Group 1 subjects had completed the study procedures up to 30 days after booster vaccination (Visit B30) and their safety data had been reviewed. An interim analysis was performed on each subject group at 90 days following the last study vaccination. The study was then unblinded, and any placebo subjects still active in the study were no longer required to follow-up. Subjects assigned to AGMG0201 groups continued in the study as per the schedule of assessments, with further follow-up Visits B180 and B360 (end-of-study visit).

AGMG0201 includes 0.2 mg of plasmid DNA, 0.25 mg (low dose) or 0.5 mg (high dose) of AngII-KLH, and 1.25 mg of Adju-Phos. A saline solution was used for the placebo. Each subject received a single injection to the deltoid muscle at Visit V0, followed by a second injection to the deltoid muscle of the same treatment administered at Visit V0, in the same arm unless otherwise not possible, approximately 30 days later at Visit B0. At each dose administration visit, subjects remained in the clinic for 24 hours after vaccination for safety monitoring. Subjects attended outpatient visits for study procedures at 7, 14, and 30 days after the first dose and at 7, 14, 30, 60, 90, 180, and 360 days after the booster vaccination. The schedule of screening and treatment has been shown in Supplementary Fig. S1.

### Enzyme-linked immunosorbent assay of human sera

Anti-angiotensin II antibody concentrations were measured as antibody titres with an angiotensin II-specific ELISA described previously [5–9]. For the ELISA of human sera, a responder was defined as a subject whose titer was above a cutoff titer. One participant (placebo group) disagreed with this measurement.

## Results and conclusion

**Fig. 1:**
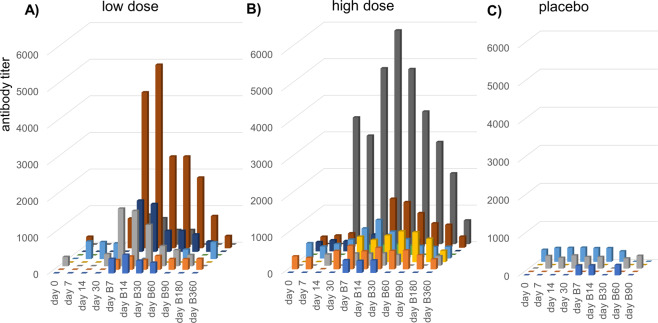
Anti-angiotensin II antibody titer. The antibody titer is shown in (**A**) the high-dose group (*N* = 9), (**B**) the low dose group (*N* = 9), and (**C**) the placebo group (*N* = 5). The antibody titer was measured at 0, 7, 14, and 30 days after the first vaccination with AGMG0201 and 7, 14, 30, 60, 90, 180, and 360 days after the second vaccination (B7, B14, B30, B60, B90, B180, and B360), excluding B180 and B360 in the placebo group

In the safety evaluation, there were no severe AEs and most treatment-emergent adverse events (TEAEs) were classified as mild or moderate in severity (Table 1). One TEAE was classified as severe (back pain, unrelated), and 6 TEAEs of moderate severity were deemed related to the study treatment (feeling hot, headache, injection site erythema, injection site pain). The pain and erythema at the injection site, classified as skin and subcutaneous tissue disorders in Table 1, were reported mainly in subjects who received AGMG0201, with no dose-related trend. Anti-angiotensin II antibodies were observed, especially in the high-dose group and to a lesser extent in the low-dose group (Fig. 1). A measurable antibody titer was detected in all 9 AGMG0201 subjects of the high-dose group at Visits B7, B14, and B30 following the second dose. In the low-dose group, eight of nine AGMG0201 subjects had measurable levels at Visit B30, and 6/9 of them had measurable levels at Visits B7, B14, and B60. Interestingly, sustained antibody levels of six subjects in the high-dose group were observed at Visit B360. Two subjects in each group had high anti-angiotensin II antibody titers (>5000); however, there were large individual differences in antibody titer in both the low-dose and high-dose groups. Overall, AGMG0201 at both low and high doses was well tolerated in subjects with mild to moderate essential hypertension.

**Table 1:** Summary of treatment-emergent adverse events related to study drug for total period

	Low *N* = 9	High *N* = 9	Placebo *N* = 6	Total *N* = 24
Subjects with at least one related TEAE	9 (100%)	4 (44%)	4 (67%)	17 (71%)
Nervous system disorders	5 (56%)	0	2 (33%)	7 (29%)
Dizziness	1 (11%)	0	0	1 (4%)
Head discomfort	1 (11%)	0	0	1 (4%)
Headache	5 (56%)	0	2 (33%)	7 (29%)
Cardiac disorders	0	0	1 (17%)	1 (4%)
Palpitations	0	0	1 (17%)	1 (4%)
Respiratory, thoracic and mediastinal disorders	1 (11%)	0	0	1 (4%)
Cough	1 (11%)	0	0	1 (4%)
Gastrointestinal disorders	2 (22%)	0	1 (17%)	3 (13%)
Abdominal distension	1 (11%)	0	0	1 (4%)
Abdominal pain	1 (11%)	0	0	1 (4%)
Bowel movement irregularity	0	0	1 (17%)	1 (4%)
Diarrhea	1 (11%)	0	0	1 (4%)
Nausea	1 (11%)	0	1 (17%)	2 (8%)
Vomiting	1 (11%)	0	1 (17%)	2 (8%)
General disorders and administration site conditions	8 (89%)	4 (44%)	3 (50%)	15 (63%)
Feeling hot	1 (11%)	0	0	1 (4%)
Injection site bruising	0	0	1 (17%)	1 (4%)
Injection site erythema	1 (11%)	2 (22%)	0	3 (13%)
Injection site mass	0	1 (11%)	0	1 (4%)
Injection site pain	8 (89%)	4 (44%)	1 (17%)	13 (54%)
Injection site pruritus	1 (11%)	0	0	1 (4%)
Injection site swelling	0	0	1 (17%)	1 (4%)

Total period means V0 to B360 (360 days after the second dose) excluding Placebo after B90

*Low* low dose of AGMG0201, *High* high dose of AGMG0201, Placebo Saline

**Fig. 2:**
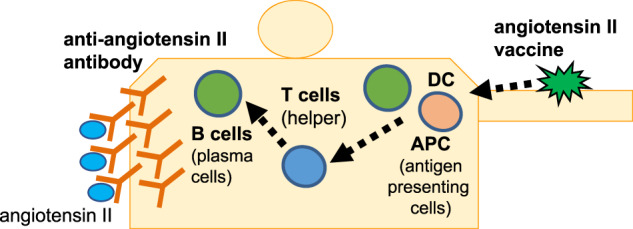
Graphical Abstract: We conducted a placebo-controlled dose escalation study (first-in human study) to investigate the safety, tolerability and immunological responses of the angiotensin II vaccine (AGMG0201)

Although this exploratory study had a limited sample size, the potential immunological reaction to AGMG0201 was observed. The nature of this study made it difficult to evaluate the clinical efficacy of AGMG0201 in terms of blood pressure change because the hypertensive responders to renin-angiotensin blockade were excluded by the screening of their tolerance to the highest dose of ARB/ACEi (the run-in period of 14–28 days) in order to protect the safety of the participants. Further clinical trials will be needed to evaluate the clinical and immunological efficacy and safety of this vaccine in hypertensive patients. Although several issues still need to be investigated, we believe that therapeutic vaccines will contribute to improving the health of hypertension patients and others in the future (Fig 2).

## Supplementary information

Supplementary Table S1

Supplementary Fig. S1
