# Supplementary material for: Brief report on a phase I/IIa study to assess the safety, tolerability, and immune response of AGMG0201 in patients with essential hypertension
Source: Hypertens Res. 2021 Oct 17;45(1):61–5. doi: 10.1038/s41440-021-00755-6 (PMC8668431; doi:10.1038/s41440-021-00755-6)
Supplement: Supplementary file 1 — Supplementary Table S1 [file 41440_2021_755_MOESM1_ESM.pdf]

Supplementary Table S1 Characteristics of the participants

Characteristics	Low<br>N=9	High<br>N=9	Placebo<br>N=6	Total<br>N=24
Age (years) mean	65.7	60.9	68.7	64.6
(SD)	(7.6)	(11.8)	(6.2)	(9.3)
Sex [female; n (%)]	4 (44%)	2 (22%)	0 (0%)	6 (25%)
Ethnicity [n (%)]				
Hispanic or Latino	0 (0%)	0 (0%)	1 (17%)	1 (4%)
Race [n (%)]				
Asian	2 (22%)	1 (11%)	1 (17%)	4 (17%)
White	7 (78%)	8 (89%)	5 (83%)	20 (83%)
Height (cm) mean	168.9	176.3	177.2	173.8
(SD)	(11.3)	(10.1)	(7.9)	(10.4)
Weight (kg) mean	80.52	86.57	86.90	84.38
(SD)	(11.39)	(10.80)	(5.70)	(10.10)
BMI (kg/m^2) mean	28.26	27.89	27.78	28.00
(SD)	(3.40)	(3.24)	(2.36)	(2.99)

Low: low dose of AGMG0201; High: high dose of AGMG0201; Placebo: Saline
