# Supplementary material for: Brief report on a phase I/IIa study to assess the safety, tolerability, and immune response of AGMG0201 in patients with essential hypertension
Source: Hypertens Res. 2021 Oct 17;45(1):61–5. doi: 10.1038/s41440-021-00755-6 (PMC8668431; doi:10.1038/s41440-021-00755-6)
Supplement: Supplementary file 2 — Supplementary Fig. S1 [file 41440_2021_755_MOESM2_ESM.pdf]

Supplementary Fig.S1

All participants	6-8 days after starting anti-hypertensive medications	≥ 14 days after starting anti-hypertensive medications	6-8 days after Run-in visit 1	≥ 14 days after Run-in visit 1	≥ 14 days after start of washout, ≥ 7 days prior to V0	Days since vaccination (V0)				Days since booster (B0)							
						1	7	14	30	1	7	14	30	60	90 Key open	180	360
Screen1	Safety call 1[1]	Run-in visit 1[1]	Safety call 2[2]	Run-in visit 2[2]	Screen2	V1	V7	V14	B0	B1	B7	B14	B30	B60	B90	B180	B360

[1]. Only for those who, at Screen1, were not on a full dose of ACEi/ARB or were on only a half dose ACEi/ARB.

[2]. Only for those who, at Screen1, were not on a half dose or full dose of ACEi/ARB.
